# Does type 1 diabetes mellitus affect Achilles tendon response to a 10 km run? A case control study

**DOI:** 10.1186/s12891-015-0803-z

**Published:** 2015-11-10

**Authors:** Andrea M. Y. Wong, Sean I. Docking, Jill L. Cook, James E. Gaida

**Affiliations:** Department of Physiotherapy, Monash University, Frankston, 3199 VIC Australia; Australian Centre for Research into Injury in Sport and its Prevention (ACRISP), Federation University, Ballarat, Australia; School of Allied Health, La Trobe University, Melbourne, Australia; University of Canberra Research Institute for Sport and Exercise (UCRISE), Canberra, Australia; Discipline of Physiotherapy, University of Canberra, Canberra, Australia

**Keywords:** Exercise, Imaging, T1DM, Achilles, Tendon

## Abstract

**Background:**

Achilles tendon structure deteriorates 2-days after maximal loading in elite athletes. The load-response behaviour of tendons may be altered in type 1 diabetes mellitus (T1DM) as hyperglycaemia accelerates collagen cross-linking. This study compared Achilles tendon load-response in participants with T1DM and controls.

**Methods:**

Achilles tendon structure was quantified at day-0, day-2 and day-4 after a 10 km run. Ultrasound tissue characterisation (UTC) measures tendon structural integrity by classifying pixels as echo-type I, II, III or IV. Echo-type I has the most aligned collagen fibrils and IV has the least.

**Results:**

Participants were 7 individuals with T1DM and 10 controls. All regularly ran distances greater than 5 km and VISA-A scores indicated good tendon function (T1DM = 94 ± 11, control = 94 ± 10). There were no diabetic complications and HbA1c was 8.7 ± 2.6 mmol/mol for T1DM and 5.3 ± 0.4 mmol/mol for control groups. Baseline tendon structure was similar in T1DM and control groups – UTC echo-types (I-IV) and anterior-posterior thickness were all *p* > 0.05. No response to load was seen in either T1DM or control group over the 4-days post exercise.

**Conclusion:**

Active individuals with T1DM do not have a heightened Achilles tendon response to load, which suggests no increased risk of tendon injury. We cannot extrapolate these findings to sedentary individuals with T1DM.

## Background

Diabetes mellitus (DM) is a group of chronic metabolic disorders characterised by inappropriate levels and/or utilisation of the hormone insulin, leading to elevated blood glucose concentrations. Hyperglycaemia contributes to long-term health complications that increase morbidity and mortality [1, 2]. Type 1 diabetes mellitus (T1DM) is the result of a complex autoimmune response that destroys insulin-producing β-cells in the pancreas.

Individuals with T1DM use exogenous insulin injections to maintain glycaemic control [3]. Glycaemic control can be measured through haemoglobin A1c (HbA1c), which is generated by glucose mediated cross-linking of normal haemoglobin. Cross-linking occurs at a faster rate at high glucose levels, and therefore, HbA1c is a useful measure of glycaemic control that reflects average blood glucose concentrations over the previous 2–3 months [4, 5]. Maintaining tight glucose control to achieve HbA1c levels less than 7 % (53 mmol/mol) decreases the risk of microvascular and macrovascular complications of diabetes such a retinopathy, nephropathy, neuropathy and macrovascular complications [6–10].

Soft tissue thickening due to accelerated cross-linking of collagen is a common diabetic complication [11–13]. Tendons are susceptible to accelerated crosslinking and therefore pathological changes. This concept is supported by data from a recent systematic review, which identified greater prevalence of DM in those with tendinopathy compared to controls without tendinopathy. A higher prevalence of tendinopathy was also seen in those with DM compared to controls without DM [14]. Studies that pooled T1DM and T2DM data found a significant increase in Achilles tendon thickness in DM participants compared to controls on ultrasound imagining [15, 16], however there were no T1DM specific data.

The prevalence of T1DM among individuals with Achilles tendinopathy – based on GP diagnosis codes in electronic records – is 1.8 % (95 % confidence interval = 0 to 4.5) and therefore not significantly different from the point estimate for the Dutch population (0.8 %) [17]. Results of the systematic review found there was a complete absence of data on tendon structure in people with T1DM [18]. Understanding tendon integrity and response to load in the T1DM population is important, as physical activity is a key component of long-term metabolic control of T1DM [19] and musculoskeletal injury frequently prevents physical activity [20]. Furthermore, increased Achilles tendon thickness in conjunction with increased plantar fascia thickness and neuropathy has been associated with altered timing in onset and duration of the windlass mechanism during gait [15, 16, 21]. This can reduce the capacity of the foot to absorb shock, as it is rigid for a greater part of the gait cycle [15, 16].

This study aims to determine whether the Achilles tendon of T1DM individuals has the same response to a 10 km run compared to non-diabetic controls. This will be measured using ultrasound tissue characterisation (UTC) [22], as it can measure transient response in tendon alignment at day 2 post maximal competitive load, which then returns to baseline by day 4 [23, 24].

## Subjects

### Study design

A case–control design was used in the setting of a T1DM social running club.

### Participants

Participants were members and friends/family of HypoActive running club, a not-for-profit organisation that aims to inspire and enable individuals with T1DM to live a physically active lifestyle. Participants were excluded if they had: a previous Achilles tendon rupture; used medication known to affect tendons in the previous 3 months (e.g. fluroquinolone antibiotics, corticosteroids); inflammatory conditions such as ankylosing spondylitis, rheumatoid arthritis and related conditions; or were under the age of 18. Control participants were excluded based on the criteria above and if they had a diagnosis of diabetes mellitus (T1DM or T2DM).

## Methods

### Outcome measures

Participants had their left Achilles tendon scanned with UTC before the 10 km run, and 2 and 4 days after the run. Participants stood on an elevated box with their great toe and knee touching a wall. The tracking device (UTC tracker, UTC imaging) with a 7–10 MHz linear ultrasound probe (SmartProbe 10 L5, Terason 2000; Teratech) was placed on the posterior calcaneal region and positioned parallel to the long axis of the Achilles tendon. The data acquisition sequence captures transverse ultrasound images every 0.2 mm along 12 cm length of the tendon (UTC software, UTC imaging). The 3D data-block is assembled and UTC algorithms are used to quantify echopatterns over a rolling window of 25 continuous images (4.8 mm). The analysis was performed from the disappearance of the calcaneum to the musculotendinous junction. UTC quantifies the structural integrity of a tendon by comparing the stability of pixel brightness over contiguous transverse images and classifies them into four echo-types [22]. Echo-type I reflects homogeneity of tendon fibrils within the tendon matrix, and echo-types II, III and IV represent increasing variability in the alignment of tendon fibrils [22]. UTC is reliable in both equine [23] and human [24] tendons and has been validated against pathological tendons histologically [25–27]. The minimum detectable difference in the Achilles tendon is 0.9 % for echo-type I, 0.9 % for echo-type II, 0.3 % for echo-type III and 0.6 % for echo-type IV [24].

All UTC scans were conducted by one investigator (AW). A second investigator (SD) relabelled the scan data using random numbers so that the investigator performing the analysis (AW) was blinded to group and time. In addition, anterior-posterior (AP) diameter was calculated 2 cm proximal from the disappearance of the calcaneum.

All participants were tested for blood glucose level (BGL) using a glucometer (Optimum Xceed, Abbott Diabetes Care Inc., Alameda, CA, USA), and for HbA1c using a batch-validated HbA1c test cartridge (DCA Vantage, Siemens Medical Solutions Diagnostics, Tarrytown, NY, USA) [28].

Each participant completed a questionnaire on current and past medical history, physical activity level and tendon pain and/or injury history. The questionnaire also obtained information on the covariates of age, gender, duration of diabetes and diabetic complications. Participants also completed the VISA-A questionnaire, which provides an index of the severity of Achilles tendon pain and function on a scale of 0–100, where 100 indicates no pain or loss of function [29]. The VISA-A questionnaire has been shown to be reliable with good test-retest (*r* = 0.93), intra-rater (3 test, *r* = 0.90) and inter-rater (*r* = 0.90) reliability, as well as good stability when compared one week apart (*r* = 0.81) [29].

Height (nearest 0.1 cm) and body mass (nearest 0.1 kg) were measured to calculate body mass index (BMI), and waist circumference was measure to the nearest 0.1 cm. Average run speed was calculated and participants were asked to refrain from additional running during the 4-day study.

### Analyses

All four UTC echo-types were analysed for normality using a Kolmogorov-Smirnov test. As the UTC data were not normally distributed, the analysis used non-parametric statistics and data are reported as median and interquartile range. Differences in the four echo-types and AP diameter between the two groups at day 0 were determined using an independent-samples Mann–Whitney *U* test. Differences across day 0, 2 and 4 medians were analysed within each group using a related-samples Friedman’s test.

Spearman’s correlation was used to determine whether an association existed between proxy measures of glycaemic control (BGL and HbA1c) and echo-type I (day 0). Analysis was limited to echo-type I in order to limit the possibility of type 1 statistical errors. All analyses were performed using statistical software (IBM SPSS Statistics version 20), with an alpha level set at 0.05.

### Ethics

Ethics approval was obtained by the Monash University Human Research Ethics Committee (MUHREC) (CF11/3089-2011001741). All participants provided written informed consent prior to participating in the study.

## Results

Seven T1DM participants (5 men, 2 women; mean ± SD age 37.9 ± 7.0 years) and ten control participants (4 men, 6 women; mean ± SD age 32.9 ± 9.9 years) were included in the study. All participants regularly ran ≥5 km in a recreational capacity with a mean weekly run distance of 23 ± 19 km in the T1DM group and 15 ± 7 km in the control group (*p* = 0.24). The T1DM group had significantly higher BMI, mean BGL and HbA1c than the control group but was matched for all other variables (Table 1). No participants had a diagnosis of retinopathy, nephropathy or neuropathy. Control participants had no first degree relatives diagnosed with T1DM.

**Table 1:** Participant Characteristics

Characteristic	T1DM	Control
	(Mean ± SD)	(Mean ± SD)
Gender	5 men, 2 women	4 men, 6 women
Age (years)	37.9 ± 7.0	32.9 ± 9.9
Height (cm)	170.9 ± 8.1	171.2 ± 8.2
Body mass (kg)	74.1 ± 10.8	65.7 ± 11.9
BMI	25.4 ± 3.3*	22.2 ± 2.3
Waist circumference (cm)	85.7 ± 10.9	80.0 ± 10.0
Mean blood glucose (mmol/L)	10.1 ± 4.3*	5.1 ± 0.8
HbA1c (%)	8.7 ± 2.6*	5.3 ± 0.4
mmol/mol	71.3 ± 4.7*	34.9 ± 0.4 %
Average run speed (km/h)	10.9 ± 1.7	10.7 ± 1.5
VISA-A score	94.1 ± 9.6	93.9 ± 11.4
Avg run distance per week (km)	22.6 ± 18.9	14.8 ± 6.6

*p-value <0.05

**Fig. 1:**
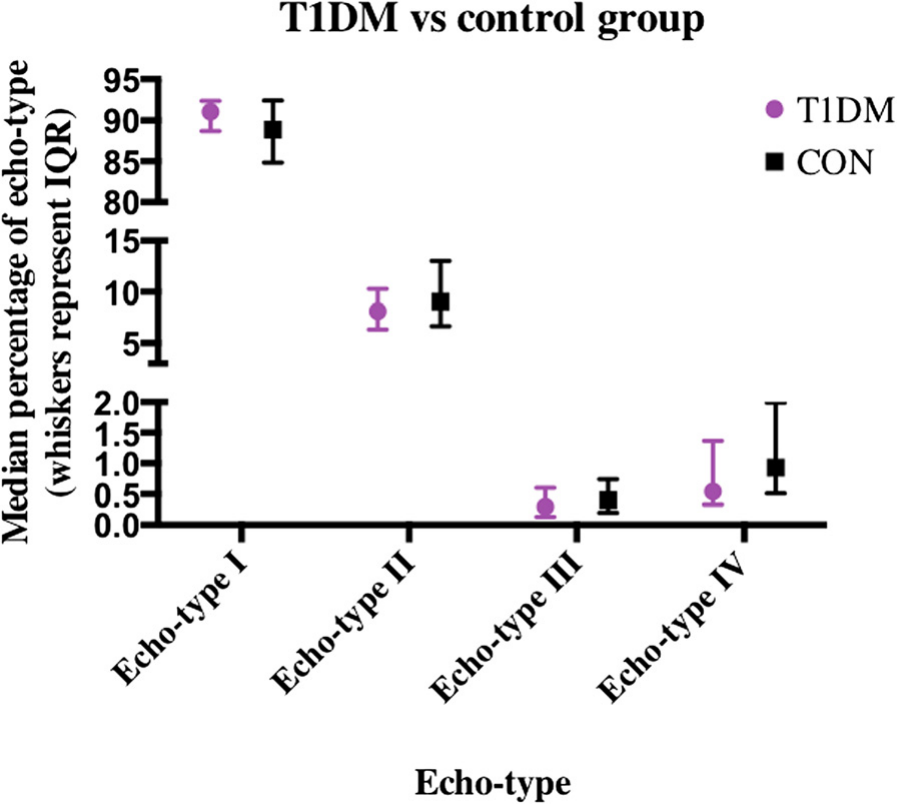
Echo-types I-IV in T1DM and control group at Day 0 (median ± IQR)

**Fig. 2:**
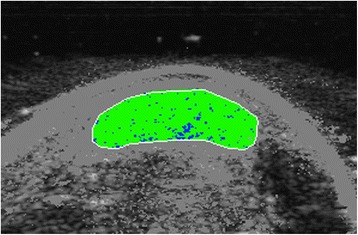
UTC image of a T1DM Achilles tendon in transverse view at baseline (Day 0). The border of the Achilles tendon is demarcated by the white line, with the surrounding pixels greyed out. Echo-types I, II, III and IV are represented as green, blue, red and black respectively

**Fig. 3:**
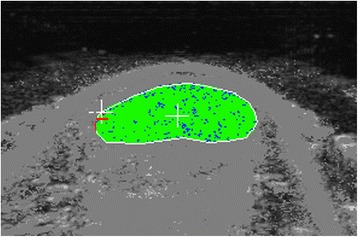
UTC image of a control Achilles tendon in transverse view at baseline (Day 0). The border of the Achilles tendon is demarcated by the white line, with the surrounding pixels greyed out. Echo-types I, II, III and IV are represented as green, blue, red and black respectively

No significant differences were observed between the groups on day 0 for echo-types I, II, III or IV (*p* = 0.313, 0.562, 0.492, 0.368 respectively, Fig. 1). Similarly, no significant difference was observed in day 0 AP tendon thickness between the T1DM and control groups (0.51 ± 0.10 cm, 0.49 ± 0.05 cm respectively, *p* = 0.368, Figs. 2 and 3 respectively).

**Fig. 4:**
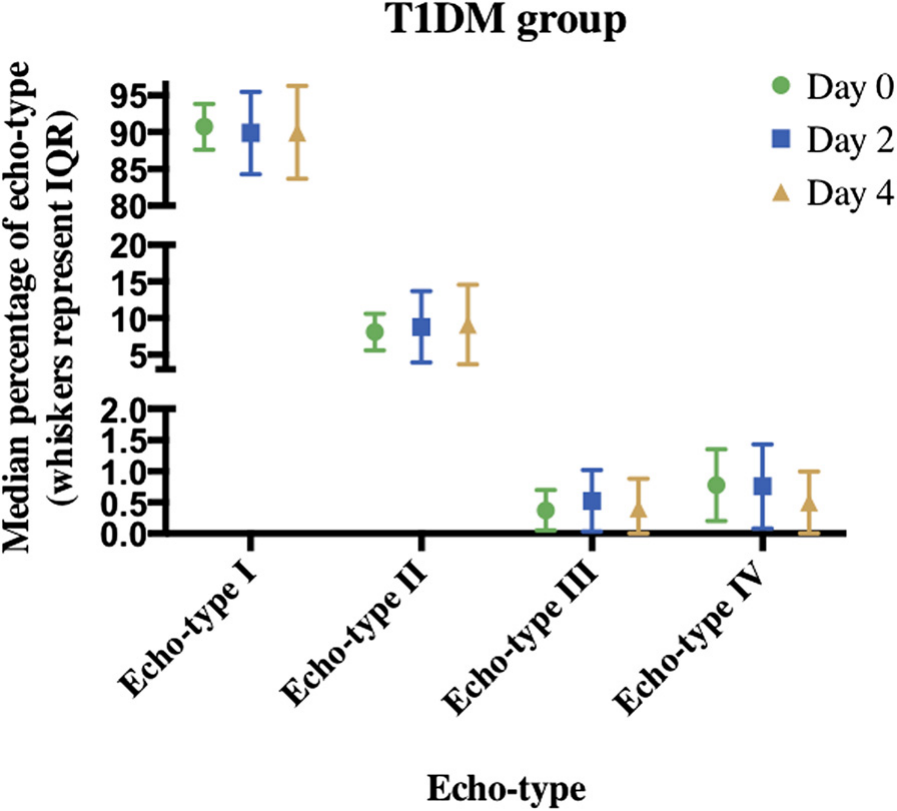
Echo-types I-IV in the T1DM group at Day 0, 2 and 4 post 10 km run (median ± IQR)

**Fig. 5:**
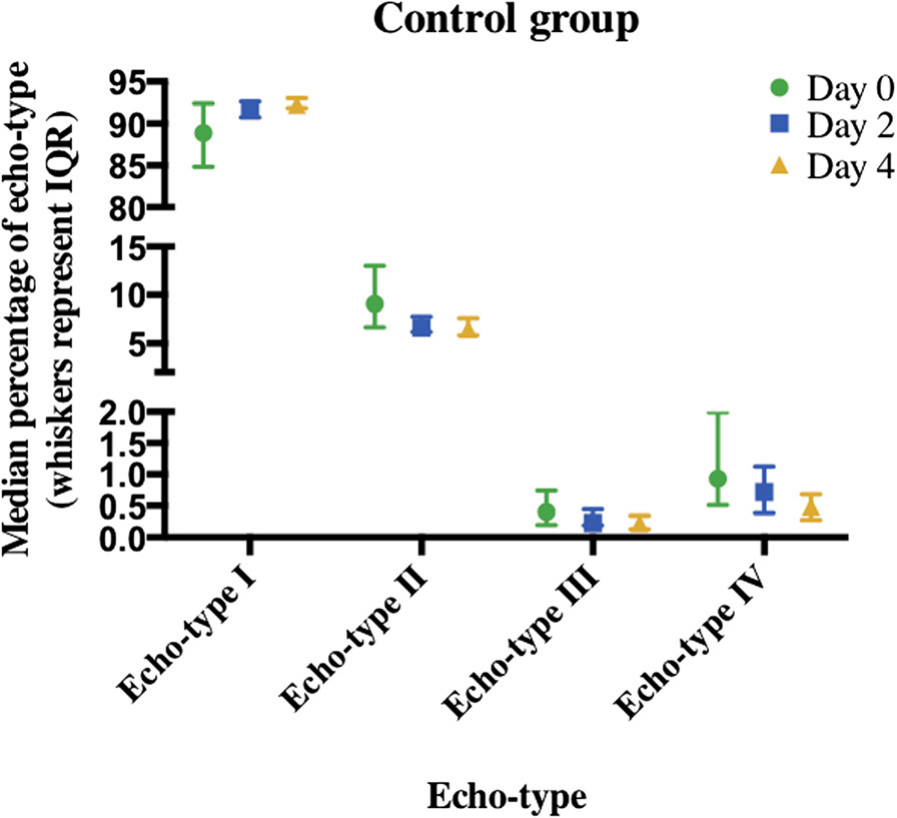
Echo-types I-IV in the control group at Day 0, 2 and 4 post 10 km run (median ± IQR)

The T1DM group had no significant differences across days in all four echo-types (I *p* = 0.368, II *p* = 1.000, III *p* = 1.000 and IV *p* = 0.174, respectively, Fig. 4). The control group similarly showed no significant differences across the four days in all four echo-types (I *p* = 0.180, II *p* = 0.156, III *p* = 0.651 and IV *p* = 0.368, respectively, Fig. 5).

Within the control group, baseline (day 0) echo-type I was not correlated with blood glucose level (Spearman’s rho = 0.13, *p* = 0.73) or HbA1c (Spearman’s rho = 0.17, *p* = 0.64). Similarly, within the T1DM group, baseline (day 0) echo-type I was not correlated with blood glucose level (Spearman’s rho = 0.50, *p* = 0.39) or HbA1c (Spearman’s rho = −0.10, *p* = 0.87).

## Discussion

This study measured Achilles tendon response to a 10 km run in individuals with T1DM and control participants. The main finding of this study was no significant change in echopattern across the four days for either group, which indicates a response was not elicited in the Achilles tendon by the 10 km run. Both the T1DM and control groups demonstrated high percentages of echo-type I, which reflects homogeneity of tendon fibrils within the tendon matrix [22].

These results differ to previous UTC studies, which found a transient response in tendons to maximal exercise at day 2 and a return to baseline by day 4 [23, 24]. These studies involved competitive sporting events that placed the tendon under maximal load, and elicited a response in the tendon that was detected using UTC. As the current study involved a recreational 10 km run rather than maximal competitive load, participants may not have maximally loaded their Achilles tendons. Many participants were completing a moderate number of kilometres per week, with some participants reporting they were currently training for a marathon. Therefore, the capacity of the Achilles tendon to tolerate load may have been much higher than was achieved during a social 10 km run. The current study also differed from previous studies as both men and women were included, and they were older than the male only participants included in the Rosengarten et al. [24] study. While no detectable response was found at 10 km for the T1DM or control groups, it is unknown whether the T1DM group would have similar or differing response in their tendons at maximal load compared to controls.

The study findings suggest T1DM individuals who are physically active have similar Achilles tendon response to controls; however tendon response in T1DM individuals who are sedentary is unknown. Achilles tendinopathy commonly affects individuals who live an active lifestyle, particularly those who are runners, however sedentary individuals are also at risk of developing the condition [30, 31]. Further research is required to determine the tendon response to load among i) sedentary individuals with T1DM, ii) individuals with T2DM who typically also have obesity, insulin resistance and elevated lipids [15, 16, 32, 33], and iii) sedentary non-diabetic individuals [34, 35]. It is important to consider this because treatment options may vary - for example, eccentric loading may be the selected treatment option for recreational athletes with a chronic Achilles tendinopathy, however may not be as beneficial in the sedentary population [35]. Sayana and Maffulli [35] found that less than 60 % of sedentary individuals had positive effects from eccentric exercise.

Another finding of the current study was that baseline (day 0) Achilles tendon structure was the same between T1DM and control groups. This concurs with several studies that found DM tendons were similar to controls or the general population. de Jonge et al. [17] reported the prevalence of T1DM in the mid-Achilles tendinopathy population was the same as the general Dutch population. Similarly, tendon structure of the flexor hallucis longus tendon on CT imaging showed no significant difference in tendon thickness between DM and control participants [36]. However, this study pooled T1/T2DM data, therefore the influence of T1DM on tendon structure is unclear.

In contrast, several studies that pooled T1/T2DM data found a significant increase in Achilles tendon thickness in DM participants compared to controls on ultrasound imaging [15, 16]. This finding was consistent across DM participants without neuropathy, DM participants with neuropathy and DM participants with neuropathic ulcers compared to controls [15, 16]. Furthermore, studies that provide T2DM only data have shown a significant increase in tendon thickness in women but not men [32], and a significant increase in tendon volume in both men and women [33].

The interest in investigating T1DM separately to T2DM comes from the profound differences in the pathophysiological causes of T1/T2 DM and the resulting exposure to hyperglycaemia. While chronic hyperglycaemia has been hypothesised to be the cause of pathological changes in tendons [12, 13], there are characteristics specific to T2DM that may predispose tendons to pathological changes. These characteristics include insulin resistance [37], elevated lipids [38, 39] and elevated adiposity [40], all of which are associated with T2DM but not commonly with T1DM. Another difference between T1DM and T2DM is time to diagnosis; T1DM is rapidly diagnosed due to severity of symptoms, whereas T2DM may have a long asymptomatic duration before diagnosis [1]. Interestingly, the years prior to T2DM diagnosis are associated with higher medical costs [41] and increased incidence of musculoskeletal conditions such as carpal tunnel syndrome [42]. Based on these differences, it is essential that future research report T1DM and T2DM data separately.

As HbA1c has been used to predict likelihood of developing diabetic complications such as retinopathy, nephropathy and neuropathy [6, 7], we analysed whether echo-type I (day 0) was correlated with HbA1c in the T1DM group. Within the T1DM group baseline (day 0) echo-type I was not correlated with blood glucose level (Spearman’s rho = 0.50, *p* = 0.39) or HbA1c (Spearman’s rho = −0.10, *p* = 0.87), however it is difficult to provide a definitive answer with a limited sample size.

Maintaining a HbA1c <7 % (53 mmol/mol) indicates good T1DM control and can decrease the likelihood of developing diabetic complications [6, 7]. However, a retrospective cohort study of 386 participants identified that only 3.4 % of T1DM individuals actually achieve this target HbA1c level, and the average T1DM HbA1c is 9.2 % [43]. Our T1DM group had a HbA1c of 8.7 % (71.3 mmol/mol) which is higher than the recommended value but below the HbA1c level in other studies. A population based study found that >50 % T1DM individuals will develop detectable diabetic complications on average 12 years after diagnosis of the disease, despite modern advancement with insulin treatment [44]. Our T1DM group were diagnosed 13 ± 12 years ago and yet none had a diagnosis of retinopathy, neuropathy or nephropathy. This complication rate is much lower than other studies with similar populations. These observations indicate we studied a highly selected sub-group of T1DM individuals who have good T1DM management, are well organised, and conscientious enough to run on a regular basis and volunteer for research projects.

It is also of interest to note that the T1DM group demonstrated larger variability in echopattern (larger IQR) compared to the control group, particularly noticeable for echo type I and II at day-2 and day-4 post run. We can speculate that this might reflect an underlying difference in the way that the Achilles tendon of individuals with T1DM responds to a bolus of load. For example, it is known that hyperglycaemia reduces proteoglycan levels and increases matrix metalloproteinase levels in cultured tendon cells [45, 46]. Whether these cell-culture findings translate to clinical observations remains unknown at present. The variability in echo type may also reflect differences in participant behaviour after the run, for example, it is unknown whether all participants refrained from physical activity for 4-days after the run as requested.

### Limitations

Limitations of this preliminary study were the small sample size and a target population of T1DM individuals with a low BMI who regularly ran in a recreational capacity. Therefore, the findings of this study cannot at present be generalised to the wider T1DM population. Furthermore, although non-significant, the T1DM group ran on average longer distances per week than control group, which may have impacted on the results of this study. Another limitation of the study was the non-competitive nature of the 10 km run, rather than a maximal competitive load.

Due to the low participant numbers, bivariate correlations were kept to a minimum. We decided to focus on BGL and HbA1c, as these were significantly different between the T1DM and control group and are key measures of T1DM control. Future studies should consider adjusting the data for covariates, such as duration of DM and HbA1c.

### Perspective

As physical activity is a key component of long-term metabolic control in T1DM [19], it is important to know how the Achilles tendon responds to load. In doing so, a better understanding and improved exercise prescription and injury management in the T1DM community can be achieved. Our findings suggest that individuals with T1DM who are regularly physically active do not have pathological changes to their Achilles tendons, in contrast to prior findings among individuals with T2DM [14]. Further research is required to determine whether our findings are unique to T1DM individuals who are regularly physically active, or whether they also apply to sedentary individuals with T1DM.

## Conclusion

We found that Achilles tendon baseline structure and response to a 10 km run over 4-days was the same in controls and T1DM individuals. The contrast with previous studies in T2DM most likely reflects the profound differences between the pathophysiology of T1DM and T2DM. This contrast strengthens the argument that tendon health of individuals with T1DM and T2DM should be studied separately.

### Ethical statement

All human studies have been approved by appropriate ethics committee as outlined in the methods, and therefore been performed in accordance with the ethical standards laid down in the 1964 Declaration of Helsinki and its later amendments.

All persons gave their informed consent prior to their inclusion in the study.

## Abbreviations

AP: Anterior-posterior
BGL: Blood glucose level
BMI: Body mass index
HbA1c: Haemoglobin A1c
SD: Standard deviation
T1DM: Type 1 diabetes mellitus
T2DM: Type 2 diabetes mellitus
UTC: Ultrasound tissue characterisation
IQR: Interquartile range
